# Classification of Cognitive Impairment and Healthy Controls Based on Transcranial Magnetic Stimulation Evoked Potentials

**DOI:** 10.3389/fnagi.2021.804384

**Published:** 2021-12-24

**Authors:** Jiahao Zhang, Haifeng Lu, Lin Zhu, Huixia Ren, Ge Dang, Xiaolin Su, Xiaoyong Lan, Xin Jiang, Xu Zhang, Jiansong Feng, Xue Shi, Taihong Wang, Xiping Hu, Yi Guo

**Affiliations:** ^1^Department of Electrical and Electronic Engineering, Southern University of Science and Technology, Shenzhen, China; ^2^School of Microelectronics, Southern University of Science and Technology, Shenzhen, China; ^3^Gansu Provincial Key Laboratory of Wearable Computing, School of Information Science and Engineering, Lanzhou University, Lanzhou, China; ^4^Department of Neurology, Shenzhen People's Hospital (The First Affiliated Hospital of Southern University of Science and Technology, The Second Clinical Medical College of Jinan University), Shenzhen, China; ^5^Department of Neurology, The Second Clinical Medical College, Jinan University (Shenzhen People's Hospital), Shenzhen, China; ^6^The First Affiliated Hospital, Jinan University, Guangzhou, China; ^7^Department of Geratic, Shenzhen People's Hospital (The First Affiliated Hospital of Southern University of Science and Technology, The Second Clinical Medical College of Jinan University), Shenzhen, China; ^8^School of Intelligent Systems Engineering, Sun Yat-sen University, Shenzhen, China; ^9^Shenzhen Bay Laboratory, Shenzhen, China

**Keywords:** spatiotemporal features, machine learning, cognitive impairment, TEP, TMS-EEG

## Abstract

**Backgrounds:** Nowadays, risks of Cognitive Impairment (CI) [highly suspected Alzheimer's disease (AD) in this study] threaten the quality of life for more older adults as the population ages. The emergence of Transcranial Magnetic Stimulation-Electroencephalogram (TMS-EEG) enables noninvasive neurophysiological investi-gation of the human cortex, which might be potentially used for CI detection.

**Objectives:** The aim of this study is to explore whether the spatiotemporal features of TMS Evoked Potentials (TEPs) could classify CI from healthy controls (HC).

**Methods:** Twenty-one patients with CI and 22 HC underwent a single-pulse TMS-EEG stimulus in which the pulses were delivered to the left dorsolateral prefrontal cortex (left DLPFC). After preprocessing, seven regions of interest (ROIs) and two most reliable TEPs' components: N100 and P200 were selected. Next, seven simple and interpretable linear features of TEPs were extracted for each region, three common machine learning algorithms including Support Vector Machine (SVM), Random Forest (RF), and K-Nearest Neighbor (KNN) were used to detect CI. Meanwhile, data augmentation and voting strategy were used for a more robust model. Finally, the performance differences of features in classifiers and their contributions were investigated.

**Results:** 1. In the time domain, the features of N100 had the best performance in the SVM classifier, with an accuracy of 88.37%. 2. In the aspect of spatiality, the features of the right frontal region and left parietal region had the best performance in the SVM classifier, with an accuracy of 83.72%. 3. The Local Mean Field Power (LMFP), Average Value (AVG), Latency and Amplitude contributed most in classification.

**Conclusions:** The TEPs induced by TMS over the left DLPFC has significant differences spatially and temporally between CI and HC. Machine learning based on the spatiotemporal features of TEPs have the ability to separate the CI and HC which suggest that TEPs has potential as non-invasive biomarkers for CI diagnosis.

## 1. Introduction

Cognitive impairment (CI) refers to a cognitive function decline beyond typical aging, which is increasingly prevalent in the elderly and loom as a public health issue (Montine et al., 2021).

Clinically, Montreal Cognitive Assessment (MoCA) and Mini-Mental State Examination (MMSE) are commonly used for routine cognitive screening. MoCA is more sensitive than MMSE in detecting mild cognitive impairment (MCI) (Ciesielska et al., 2016). For Alzheimer's disease (AD) diagnosis, there are two main types of biomarkers: biophysiological biomarkers such as β amyloid in cerebrospinal fluid (CSF)/plasma/serum and brain imaging markers (Frisoni et al., 2017; Ng et al., 2019; Cullen et al., 2021). For example, Aβ42/Aβ40 ratio in CSF and blood (Buchhave et al., 2012; Hansson et al., 2018), Positron Emission Tomography (PET) of beta-amyloid and tau proteins (Leuzy et al., 2019; Rabinovici et al., 2019). However, the invasiveness of the collection of body fluids and high cost of PET limit their large-scale use. In recent years, some articles have reported that combining different neuroimaging modalities together can effectively detect CI. For example, combining functional MRI (fMRI) and Diffusion Tracking Imaging (DTI) can reflect functional connectivity changes in neuronal networks between CI and Healthy Controls (HC) (Ye and Bai, 2018). Multimodal fusion combines the advantages of each modality, but it is undeniable that complex data fusion algorithms impose huge challenges for clinical application.

Due to the above problems, some researchers have turned their attention to find a quick, noninvasive, and inexpensive method to detect CI, especially for mild and moderate patients without obvious behavioral symptoms. As a non-invasive, high time resolution method, the electroencephalogram (EEG) has been widely used in clinical examinations. In recent decades, the abnormalities in the resting state EEG of patients with CI have been discovered, such as a shift of the power spectrum to lower frequencies, a decrease in the coherence of fast rhythms, a decreased complexity of EEG patterns (Jeong, 2004).

With the development of non-invasive neuromodulation technology, the combination of Transcranial Magnetic Stimulation and Electroencephalogram (TMS-EEG) allows external input to specific cortical areas of subjects in a controlled and quantitative way for direct functional assessment (Hallett, 2007; Kimiskidis, 2016; Cao et al., 2021). When TMS pulses are applied to the cortex, trans-synaptic activation of local and distal cortical networks is obtained (Tremblay et al., 2019). The sum of synaptic potentials can be recorded simultaneously by high time resolution, multichannel scalp EEG. There are a series of positive and negative deflections after TMS, known as TMS Evoked Potentials (TEPs). The TEPs last 300 ms or more and can be recorded by either local or distal electrodes (Komssi et al., 2002), reflecting the spread of activation over cortical regions that are functionally connected and indicating the state of the brain further (Nikulin et al., 2003). Compared with resting-state EEG, TMS-EEG provides controlled stimulation without the involvement of the participation, which is more stable and objective.

At present, some researchers have used TMS-EEG to assess patients with CI. For example, the prefrontal TMS-evoked activity was able to track disease progression in Alzheimer's Disease (AD) and the P30 amplitude was predictive of the MMSE score in patients with AD (Bagattini et al., 2019). The Motor-Evoked Potentials (MEPs) produced by paired pulses on the primary cortex can be used as indicators in the classification of different subtypes of MCI (Benussi et al., 2021).

In this study, we hypothesized that TEPs resulting from stimulation of the left DLPFC may be associated with the cognitive status, thus, the features of TEPs could further differentiate CI and HC. On the premise of preserving time and space features simultaneously, we extracted some concise, interpretable linear features of TEPs in seven regions of interest (ROIs). We aimed to classify CI and HC automatically through machine learning based on the spatiotemporal features of TEPs and find potential biomarkers for clinical diagnosis.

## 2. Materials and Methods

**Figure 1:**
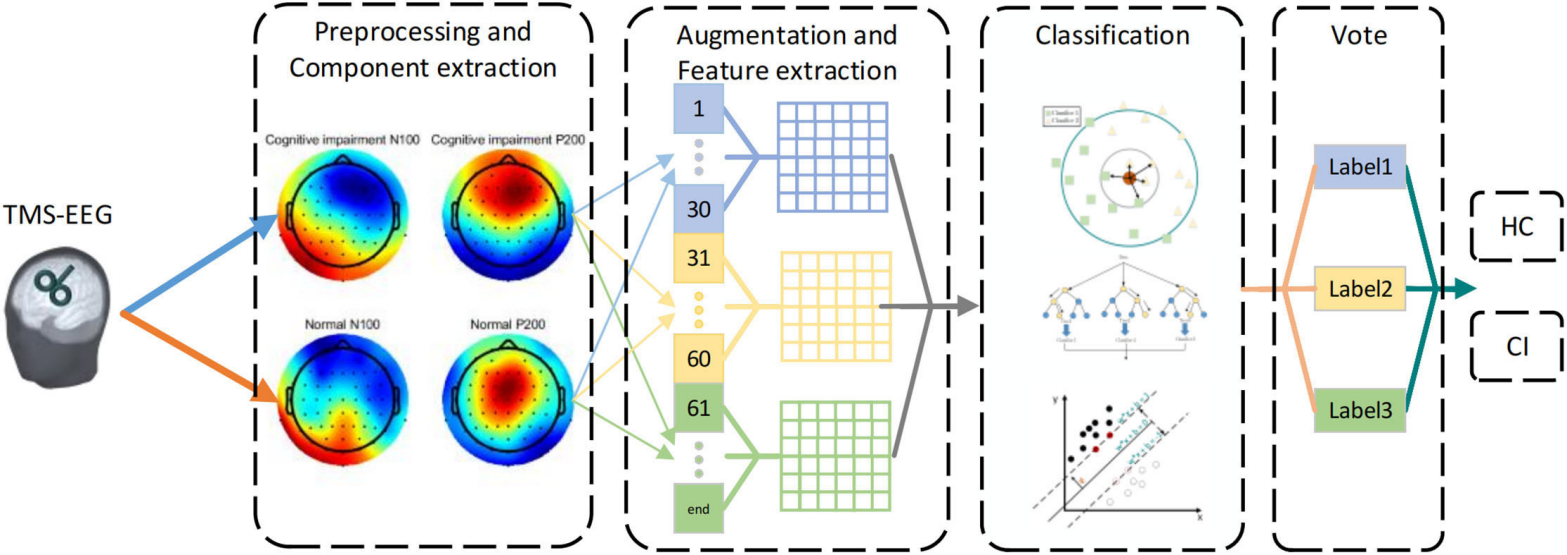
The framework of CI detection based on TEPs. First, the N100 and P200 components of TEPs were selected after removing artifacts. Then, all trials were divided into three segments. Subsequently, seven features were extracted from different segments and regions of interest (ROIs) respectively. Finally, machine learning was used to classify features, and voted on each segment to get the predicted result.

The framework is shown in Figure 1. We removed artifacts of TMS-EEG at first. Then, we divided all the trials of each participant into three segments to the augment dataset. In order to preserve the features of time and space simultaneously, we focused on the TEP's N100 and P200 components in seven ROIs. Next, we explored some concise, interpretable linear features of these two components including Local Mean Field Power (LMFP), Latency, Amplitude, Standard Deviation, Average Value (AVG), Area Under the Curve (AUC), and Range. Finally, we used three common machine learning algorithms: K-Nearest Neighbor (KNN), Support Vector Machine (SVM), and Random Forest (RF) to obtain the label of each segment, and voted to get participant's final prediction result (Cover and Hart, 1967; Vapnik, 1999; Liaw and Wiener, 2002).

### 2.1. TMS-EEG Data Acquisition

#### 2.1.1. Study Participants

All participants in this study were recruited from the Department of Neurology in Shenzhen People's Hospital (The First Affiliated Hospital, Southern University of Science and Technology). This study was approved by the Institutional Review Board of Shenzhen People's Hospital. All participants provided written informed consent.

A total of 43 subjects participated in this study, including 21 patients with CI and 22 HC. The inclusion criteria of CI group were: (a) Clinicians highly suspect the subject has AD based on the clinical diagnostic criteria (National Institute of Neurological and Communicative Disorders and stroke and the AD and Related Disorders Association (NINCDS-ADRDA), the Diagnostic and Statistical Manual of Mental Disorders-V (DSM-V) criteria); (b) Mild and moderate CI diagnosed (10 < MoCA <26) (Nasreddine et al., 2005); (c) aged 50–75 years old. The exclusion criteria were: (a) blood vessels and other types of dementia; (b) severe CI (MoCA <10); (c) a history of other psychiatric or other neurological disorders, such as schizophrenia, Parkinson's disease, and multiple sclerosis; (d) any contraindication for TMS, such as a metallic implanted device in or near the head and aneurysms.

All of the subjects in the control group met the following criteria: (a) aged 50–75 years old; (b) never complained of cognition or memory problems; (c) no history of any psychiatric or neurological disorders, brain injury, cranial neurosurgery, alcohol or drug abuse, or any severe chronic systemic illness; (d) no contraindication for TMS.

There was no significant difference in age between the two groups (*p* = 0.406). Demographic information was summarized in Table 1.

**Table 1:** Demographic subjects.

	**CI**	**HC**
Subject(s)	21	22
Age (mean ± SD)	61.86 ± 4.77	60.77 ± 3.65
Sex (male/female)	9:12	10:12
MoCA (mean ± SD)	20.33 ± 4.44	/

#### 2.1.2. TMS-EEG Recordings

The dorsolateral prefrontal cortex (DLPFC) is a key node of various cognitive functions such as memory, attention, and execution (Carlén, 2017). As we aimed to research cognition related function, the left DLPFC is also a recommended target for TMS treatment (Ahmed et al., 2012). Therefore, we chose left DLPFC to be the target of stimulation in this study.

All of the subjects in this study underwent a TMS-EEG protocol. A total of 100 single-pulse TMS pulses were delivered using the MagPro X100 with MagOption(MagVenture, Copenhagen, Denmark). The coil (figure-8 coil, Coil B65; external wing diameter, 90 mm) was placed over F3 (International 10/20 EEG system) to target the left DLPFC. The Inter-Stimulus Interval (ISI) was 3s jittered, and the stimulation intensity was 120% Resting Motor Threshold (RMT). The RMT is determined as the minimum stimulus intensity that produces a MEP exceeding 50 μV in a minimum of five out of ten trials in the relaxed right abductor pollicis brevis.

While receiving TMS, EEG signals of subjects were collected by BrainAmp DC amplifier (Brain Products, Munich, Germany) with a 64-channel EEG system. Participants were asked to remain still and relaxed during the EEG recording. The sampling rate was maintained at 5 kHz, and electrode impedances were maintained below 5 kΩ by applying the conductive gel. FCz was used as the reference while AFz was the ground during the EEG recording. All recordings took place in a temperature-controlled and electrically shielded room. Participants were asked to listen to white noise through earphones in order to mask the loud click accompanied by TMS coil discharge. A foam layer was placed under the coil to inhibit bone conduction and scalp sensation caused by the vibration of the coil (Rogasch et al., 2014).

### 2.2. TMS-EEG Data Preprocessing

#### 2.2.1. Remove Artifacts

The TMS-EEG data in this study were preprocessed offline with TMS-EEG Signal Analyser (TESA) toolbox (Rogasch et al., 2017). TESA is an open source extension for EEGLAB (Delorme and Makeig, 2004), which is used for cleaning and analyzing TMS-EEG data. Both EEGLAB and TESA ran in Matlab (R2020b).

The data were divided into two-second epochs (−1,000 to 1,000 ms, the time of stimulation was marked as 0 s) and then baseline corrected (−500 to −50 ms). In order to remove the huge electromagnetic artifacts associated with TMS, the large amplitude TMS pulse artifact was removed between −5 and 15 ms and cubic interpolation was used to replace the removed data. For more efficient computing, the sampling rate of data was reduced from 5 to 1 kHz. Epochs and channels contaminated seriously were removed during visual inspection.

The first round of Independent Component Analysis (ICA) was performed to remove large value artifacts including TMS-evoked muscle artifacts and decay artifacts. Subsequently, the data were band-pass (1–80 Hz) and band-stop (48–52 Hz) filtered. It was followed by the second round of ICA to remove other relatively small value artifacts including auditory artifacts, blinks, eye movement, persistent scalp muscle activity, and electrode noise. Finally, the missing channels removed in the preprocessing were interpolated using spherical interpolation and all channels were re-referenced to the common average (Rogasch et al., 2014).

#### 2.2.2. Time-Locked Averaging and GMFP

**Figure 2:**
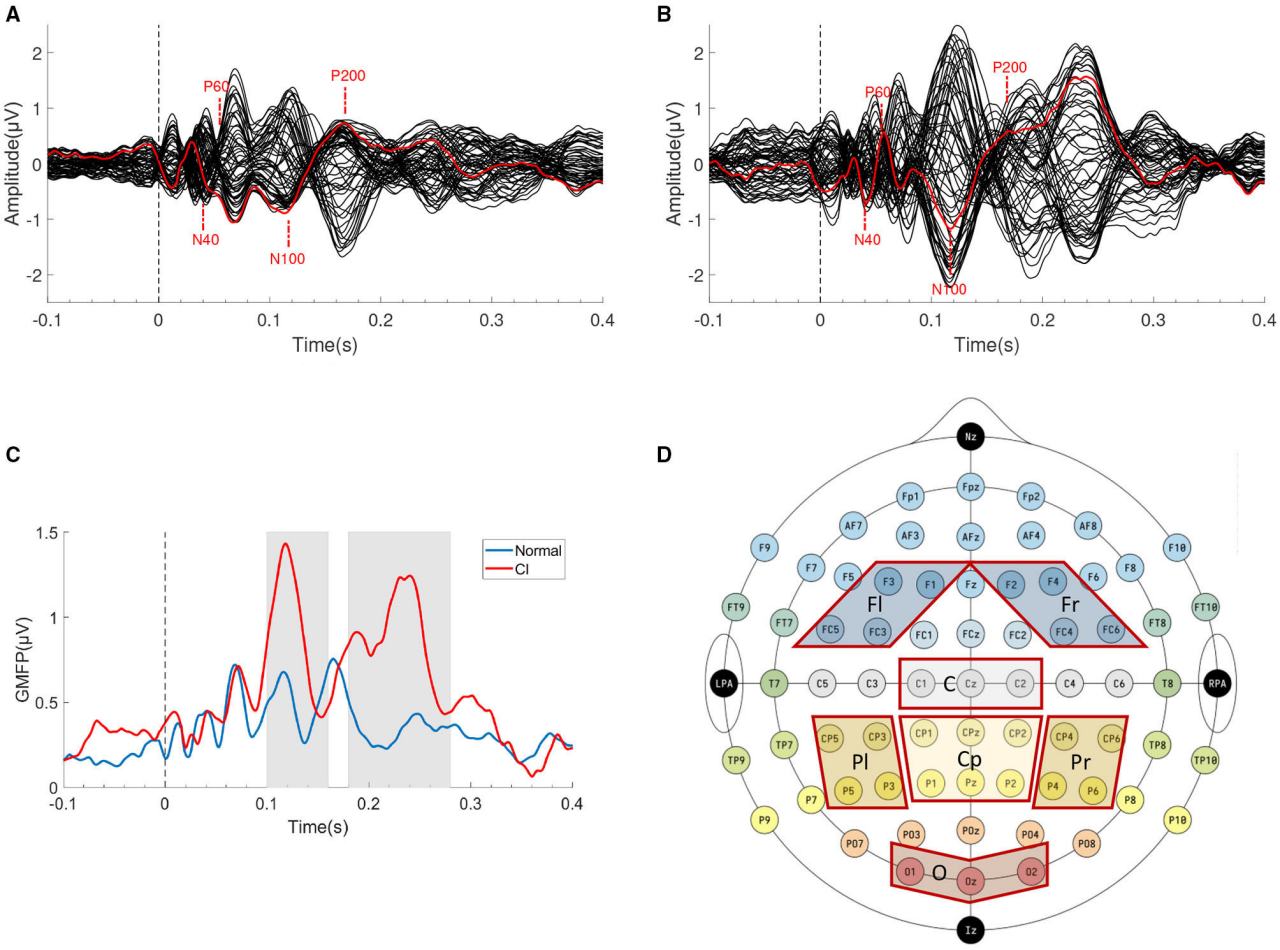
TMS Evoked Potentials (TEPs) components and ROIs selection. The grand average butterfly plot of all channels' TEPs of **(A)** HC group and **(B)** CI group. The electorde (F3) under the TMS coil is indicated in red. **(C)** The GMFP of two groups, the gray areas indicate two time windows of N100 (100–160 ms) and P200 (180–280 ms). **(D)** Schematic diagram of seven ROIs.

After data preprocessing, we got clean TMS-EEG trials (1s before stimulation, 1s after stimulation). TEPs were computed by averaging selected artifact-free single trial. The grand average TEPs of the two groups were shown in Figures 2A,B.

TMS Evoked Potentials can be recorded from the local electrode to the stimulation site, also from the electrode located in the distant cortical region. The Global Mean Field Power (GMFP) is usually calculated as a measure of global cortical excitability if ROIs are not specified. GMFP is the standard deviation (SD) of all channels at a given sampling point (Esser et al., 2006). The calculation formula was shown in Equation (1). The GMFP curve was shown in Figure 2C, which also indicated that the two groups may have differences in the two time windows of N100 and P200 (gray areas).


(1) $$\begin{array}{l} GMFP\left(i\right)=\sqrt{\frac{\sum_{j=1}^{K}{\left(V_{\text{j}}\left(i\right)-V_{\mathrm{mean}}\right)}^{2}}{K}} \end{array}$$

where K is the number of all channels (*K* = 62 in this study), *V*_*j*_(*i*) is defined as the voltage measured with channel j at sampling point i, and *V*_*mean*_ represents the average of the voltages across all channels.

#### 2.2.3. Data Augmentation and Gaussian Smoothing

In order to improve the stability of subsequent machine learning, data augmentation technique was applied. We divided all the trials (the trials remaining after artifacts removing) of each subject into three segments (1–30, 31–60, and 61-end), and then averaged the trials in each segment, which meant that each subject had three segments available for training.

The TEPs obtained by a smaller number of trials are not as smooth as all the trials. In order to make the subsequent feature extraction more reliable, especially the Latency and Amplitude, we used Gaussian window method (the length of Gaussian window is set to 20) to smooth the data after averaging (Gwosdek et al., 2011).

#### 2.2.4. TEPs' Time Windows and ROIs Selection

We found four typical characteristic peaks in Figures 2A,B which were consistent with previous research (N40, P60, N100, and P200) (Rosanova et al., 2012; Rogasch et al., 2015). In these typical peaks, N100 and P200 are widely regarded as the two most reliable and reproducible peaks (Kerwin et al., 2018). Considering individual variation led to the advancement or delay of the latency of characteristic peaks, we chose two relatively wide time windows (100–160 ms, 180–280 ms) to include peaks in the window to the full extent.

For the spatial features of TEPs, we selected 7 ROIs according to previous research (Kerwin et al., 2018). The seven ROIs are left Frontal (Fl, F1/F3/FC3/FC5), right Frontal (Fr, F2/F4/FC4/FC6), Central (C, Cz/C1/C2), Centroparietal (Cp, CPz/CP1/CP2/Pz/P1/P2), left Parietal (Pl, CP3/CP5 /P3/P5), right Parietal (Pr, CP4/CP6/P4/P6), and Occipital (O, Oz/O1/O2), as shown in Figure 2D.

### 2.3. Temporal-Spatial Features Extract

The average TEPs recorded by all channels in each ROI was calculated as the TEP of this ROI, as shown in Equation (2).


(2) $$\begin{array}{l} X\left(i\right)=\frac{1}{k}\overset{k}{\underset{j=1}{\sum }}V_{j}\left(i\right) \end{array}$$

where X(i) is TEP in the selected ROI, k is the number of channels in this ROI, *V*_*j*_(*i*) is defined as the voltage measured with channel j at the sampling point i.

In order to describe the details of the two peaks (N100 and P200) as much as possible, we calculated a series of linear features in the selected time windows and regions. The features we extracted were introduced below:

- Latency and Amplitude. The Latency and Amplitude are the most common approaches for quantifying TEP (Tremblay et al., 2019). That is, the time and amplitude of the largest peak (negative or positive).
- Local Mean Field Power. The LMFP refers to SD across specific channels in the selected ROI (i.e., electrodes of interest, EOI). We calculated at every sampling point in the given time window and then averaged them. The LMFP reflects the dispersion degree of the signals recorded by the electrodes in this region indicating local excitability changes (Pellicciari et al., 2013), as shown in Equation (3).
  (3) $$\begin{array}{l} f_{LMFP}=\frac{1}{N}\overset{N}{\underset{i=1}{\sum }}\sqrt{\frac{\sum_{j=1}^{k}{\left(V_{j}\left(i\right)-V_{\mathrm{mean}}\right)}^{2}}{k}} \end{array}$$
  where N is the number of all sampling points in the time window.
- Standard Deviation (STD). The STD is the standard deviation of the signal value in the selected time window, reflecting the degree of dispersion of the signal, as shown in Equation (4). This is an important time domain feature in EEG also called activity. The activity is quantified by means of the amplitude variance (Hjorth, 1970).
  (4) $$\begin{array}{l} f_{STD}=\sqrt{\frac{1}{N}\overset{N}{\underset{i=1}{\sum }}{\left(X\left(i\right)-\frac{1}{N}\overset{N}{\underset{i=1}{\sum }}X\left(i\right)\right)}^{2}} \end{array}$$
- Area Under Curve (AUC). The AUC is the area of the envelope between the signal and the time axis. The upper part of the time axis is positive and the lower part is negative. We used numerical integration to calculate the area, as shown in Equation (5). AUC was also called cortical-evoked activity (CEA) in previous research (Rajji et al., 2013).
  (5) $$\begin{array}{l} f_{AUC}=\frac{1}{2f}\overset{N-1}{\underset{i=1}{\sum }}\left(X\left(i\right)+X\left(i+1\right)\right) \end{array}$$
  where f is the sampling rate.
- Average Value. The AVG is the average signal value in the selected time window.
- Range. The Range is the difference between the maximum value and the minimum value of the signal in the selected time window, reflecting the fluctuation degree of the signal.

### 2.4. Machine Learning

In each ROI, we extracted 7 features of N100 and P200 respectively. Finally, 98 (2 time windows^*^7 ROIs^*^7 features) features were obtained in each segment of each subject.

Due to the possible correlation of different features, the t-Distributed Stochastic Neighbor Embedding (t-SNE) (van der Maaten and Hinton, 2008) was used to reduce the dimension. After features' dimension reduction, the feature array was normalized to [−1, 1].

In this study, three machine learning algorithms were used. SVM was implemented in the LIBSVM toolbox (Chang and Lin, 2011) with default parameters (linear kernal). Other classifiers [RF(*n*_*tree*_ = 7) and KNN(*k* = 5)] were also implemented in Matlab.

Since the features were divided into three segments, three labels that had the same weight were obtained for each participant after the classifier's prediction. We used the voting strategy to fusion three labels. The most pointed category was considered the final label of the subject.

In order to evaluate the performance of the classifiers and to simulate the reality of real CI recognition as much as possible, we adopted a leave-one-out cross-validation (LOOCV) strategy, keeping the minimum subject subset containing all the segment of a subject as the test set and employing all the others for training.

It is necessary to evaluate the classification effect of the model using appropriate indicators. For the binary classification problem, the test set can be divided into: True Positive (TP), False Positive (FP), False Negative (FN), and True Negative (TN). In this study, the subjects with CI were defined as positive samples, HC were defined as negative samples. The several evaluation indicators we used in this section are as follows:


(6) $$\begin{array}{l} Accuracy=\frac{TP+TN}{TP+TN+FP+FN} \end{array}$$

(7) $$\begin{array}{l} Sensitivity=\frac{TP}{TP+FN} \end{array}$$

(8) $$\begin{array}{l} Specificity=\frac{TN}{TN+FP} \end{array}$$

(9) $$\begin{array}{l} \text{F1-score}=\frac{2TP}{2TP+FP+FN} \end{array}$$

### 2.5. Statistics

#### 2.5.1. Cluster-Based Permutation Test

Electroencephalogram data has both time and space structure (sampled in multiple channels and multiple time points). Therefore, the difference between CI and HC was the evaluation of a very large number of channel-time pairs, which was a multiple comparisons problem (MCP). For TEPs, we used cluster-based permutation statistics at the whole scalp level to take into account any combination of space and time, while controlling the MCP (Maris and Oostenveld, 2007). We performed an independent *t*-test for the two groups in the selected time windows (100–160 ms, 180–280 ms). If the test statistic value observed in at least two adjacent channels was lower than the threshold value of 0.05, then this value was considered in the cluster arrangement. We performed 5,000 iterations of trial randomization to generate permutation distributions and controlled multiple comparisons across spaces (*P* < 0.025, two-tailed test).

## 3. Results

### 3.1. Classification Results Based on Different Time Windows

According to the section 2.2.4, we extracted features in different components of TEPs (N100 and P200). Then, we explored the performance of the classifiers by using different components' features. To reduce the dimension of the features matrix, we used t-SNE to reduce the dimension of data.

Table 2 showed the classification results. All components mean merging the features of N100 and P200 components. The best classification performance was achieved by using the features of N100. The highest accuracy of 88.37% was achieved by SVM, with a specificity of 95.45%, the sensitivity of 80.95%, and the F1-score of 87.18%. The features of all components reached slightly weaker but still reasonable classification performance. The classification results of P200 were not satisfactory, the highest accuracy was 79.07%, and other classifiers had even worse results. The sensitivity, specificity, and F1-score were also lower than the N100 in different classifiers.

**Table 2:** Classification results by all classifiers in different components.

**Component**	**Classifier**	**Accuracy**	**Sensitivity**	**Specificity**	**F1-score**
N100	KNN	0.8140	0.7619	0.8636	0.8000
	SVM	**0.8837**	**0.8095**	**0.9545**	**0.8718**
	RF	0.7674	0.7619	0.7727	0.7619
P200	KNN	0.7442	0.7143	0.7727	0.7317
	SVM	0.7907	0.7619	0.8182	0.7805
	RF	0.7442	0.6667	0.8182	0.7179
All components	KNN	0.7907	0.7143	0.8636	0.7692
	SVM	0.8372	0.7619	0.9091	0.8205
	RF	0.7907	0.7619	0.8182	0.7805

*The bold values indicate the optimal result under the same index, the same as follow*.

### 3.2. Classification Results Based on Different ROIs

Regions of interest were divided according to the section 2.2.4 and we extracted features from each ROI to explore which ROIs are more sensitive to CI. Since there were only 14 features (2 time windows^*^7 features) in each ROI, the features were directly put into the classifier after normalization without dimensionality reduction.

Table 3 showed the classification results. It could be concluded that different brain regions had great influence on the classification results, and the Fr and Pl region showed the best performance, which achieved 83.72% by SVM. Moreover, when RF was used, the features of Fr were sensitive to the distinction of positive samples, which meant that the patients with CI could be well recognized and the probability of the patients with CI being diagnosed as normal was reduced. The features of the Fl region showed slightly weaker but still reasonable classification performances. The features of the C region can distinguish negative samples well, but it was not good enough to distinguish patients with CI. The features of the Cp region, O region, and Pr region were basically unable to complete the task of distinguishing normal people from patients with CI.

**Table 3:** Classification results by all classifiers in different regions.

**Region**	**Classifier**	**Accuracy**	**Sensitivity**	**Specificity**	**F1-score**
C	KNN	0.7209	0.5714	0.8636	0.6667
	SVM	0.7209	0.6190	0.8182	0.6842
	RF	0.7674	0.7143	0.8182	0.7500
Cp	KNN	0.6977	0.5714	0.8182	0.6486
	SVM	0.5814	0.4762	0.6818	0.5263
	RF	0.7674	0.7619	0.7727	0.7619
Fl	KNN	0.7674	0.6667	0.8636	0.7368
	SVM	0.7674	0.6667	0.8636	0.7368
	RF	0.8140	**0.8095**	0.8182	0.8095
Fr	KNN	0.8140	0.6667	**0.9545**	0.7778
	SVM	**0.8372**	0.7143	**0.9545**	**0.8108**
	RF	0.8140	**0.8095**	0.8182	0.8095
O	KNN	0.6744	0.6190	0.7273	0.6500
	SVM	0.7209	0.6667	0.7727	0.7000
	RF	0.7442	**0.8095**	0.6818	0.7556
Pl	KNN	**0.8372**	0.7143	**0.9545**	**0.8108**
	SVM	**0.8372**	0.7143	**0.9545**	**0.8108**
	RF	0.7674	0.7143	0.8182	0.7500
Pr	KNN	0.5814	0.3810	0.7727	0.4706
	SVM	0.5349	0.3810	0.6818	0.4444
	RF	0.7209	0.6667	0.7727	0.7000

*The bold values indicate the optimal result under the same index, the same as follow*.

### 3.3. Statistical Results

**Figure 3:**
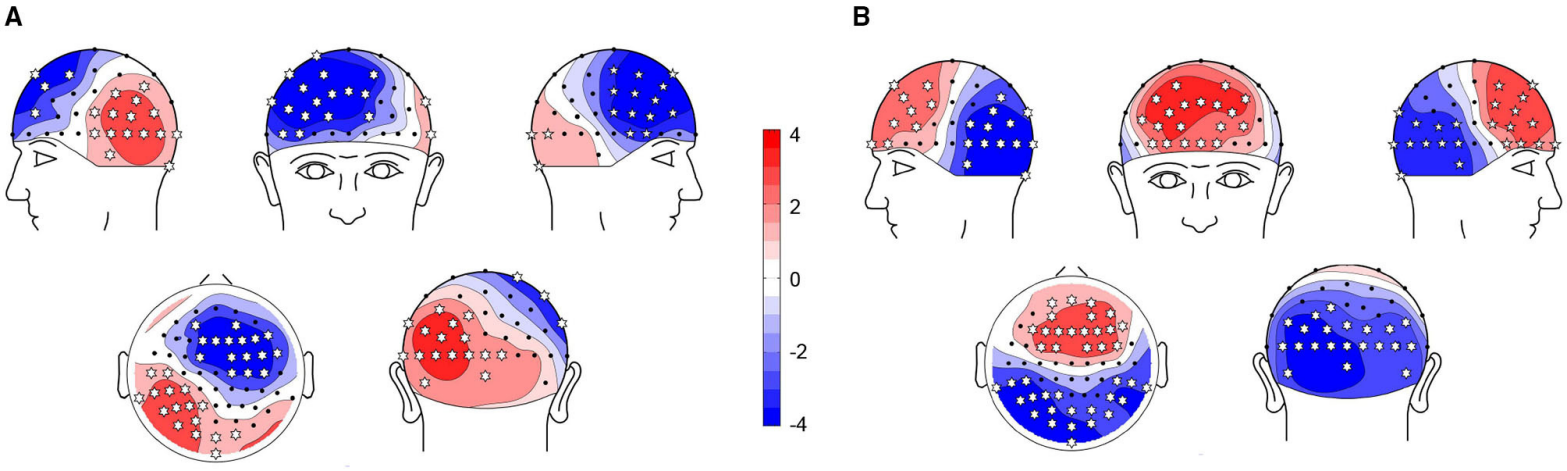
Comparison of TEPs using cluster-based permutation tests. Red means TEP of cognitive impairment (CI) is higher than HC, blue means TEP of CI is lower than healthy controls (HC). The asterisk indicates that *p* < 0.01. **(A)** N100: CI vs. HC. **(B)** P200: CI vs. HC.

We first performed a cluster-based permutation test on the TEPs of the two groups. The results of statistical analysis were shown in Figure 3. The topographic map was generated with Yang's topoplot_bcl function based on EEGLAB's topoplot function (Li et al., 2018). The asterisk indicates that the *p*-value is less than 0.01. In the N100 time window (100–160 ms), right frontal region, left parietal region, and occipital region were significantly different between the two groups (*p* < 0.01). In the P200 time window (180–280 ms), bilateral frontal region, bilateral parietal region, and occipital region were significantly different between the two groups (*p* < 0.01).

**Figure 4:**
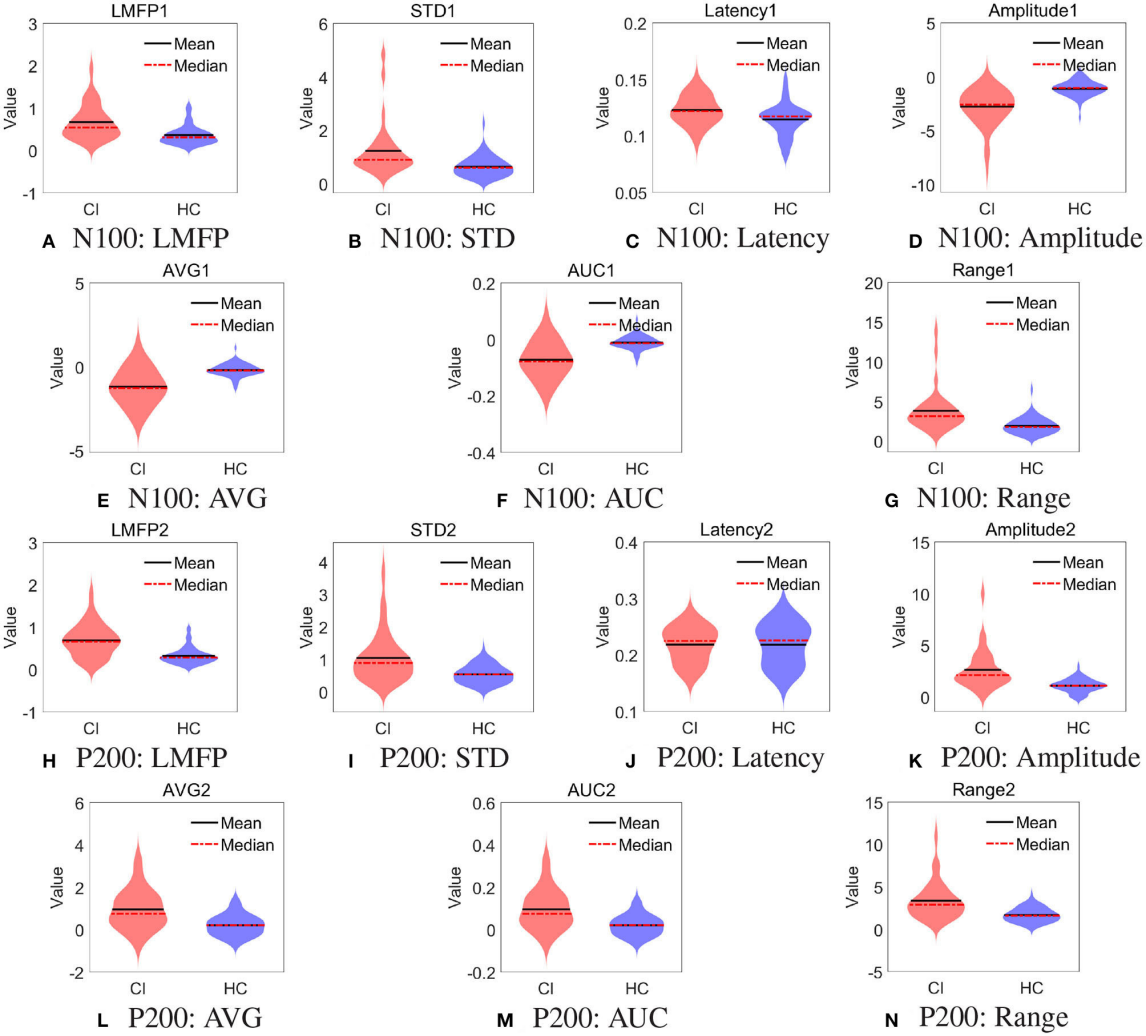
Distribution of spatial features in right frontal region. **(A)** N100: LMFP, **(B)** N100: STD, **(C)** N100: Latency, **(D)** N100: Amplitude, **(E)** N100: AVG, **(F)** N100: AUC, **(G)** N100: Range, **(H)** P200: LMFP, **(I)** P200: STD, **(J)** P200: Latency, **(K)** P200: Amplitude, **(L)** P200: AVG, **(M)** P200: AUC, and **(N)** P200: Range.

Furthermore, we used the violin plot to describe the distribution of 14 features of the right frontal region, as shown in Figure 4. We also performed *t*-test on the features in Figure 4, the results were shown in the Table 4. All the features of N100 in the right frontal region were significantly different between the two groups (*p* < 0.01). There was no difference in the latency of P200 in the right frontal region between the two groups. This may explain why the features of N100 performed better than P200 on classification, and even better than using them simultaneously.

**Table 4:** Local features in right frontal region.

	**N100**			**P200**		
	**CI**	**HC**	***P*-value**	**CI**	**HC**	***P*-value**
LMFP	0.67 ± 0.40	0.37 ± 0.22	*p* < 0.00001^*^	0.70 ± 0.38	0.33 ± 0.19	*p* < 0.00001^*^
STD	1.23 ± 0.98	0.64 ± 0.38	*p* < 0.00001^*^	1.05 ± 0.76	0.54 ± 0.27	*p* < 0.00001^*^
Latency	0.12 ± 0.013	0.11 ± 0.014	0.001^*^	0.22 ± 0.031	0.22 ± 0.036	0.961
Amplitude	–2.77 ± 1.68	–1.10 ± 0.72	*p* < 0.00001^*^	2.64 ± 1.92	1.11 ± 0.64	*p* < 0.00001^*^
AVG	–1.17 ± 1.10	–0.17 ± 0.39	*p* < 0.00001^*^	0.98 ± 0.93	0.22 ± 0.48	*p* < 0.00001^*^
AUC	–0.071 ± 0.067	–0.011 ± 0.023	*p* < 0.00001^*^	0.098 ± 0.094	0.022 ± 0.048	*p* < 0.00001^*^
Range	3.87 ± 2.71	1.97 ± 1.02	*p* < 0.00001^*^	3.38 ± 2.19	1.71 ± 0.81	*p* < 0.00001^*^

* *means a significant difference with p = 0.01*.

**Figure 5:**
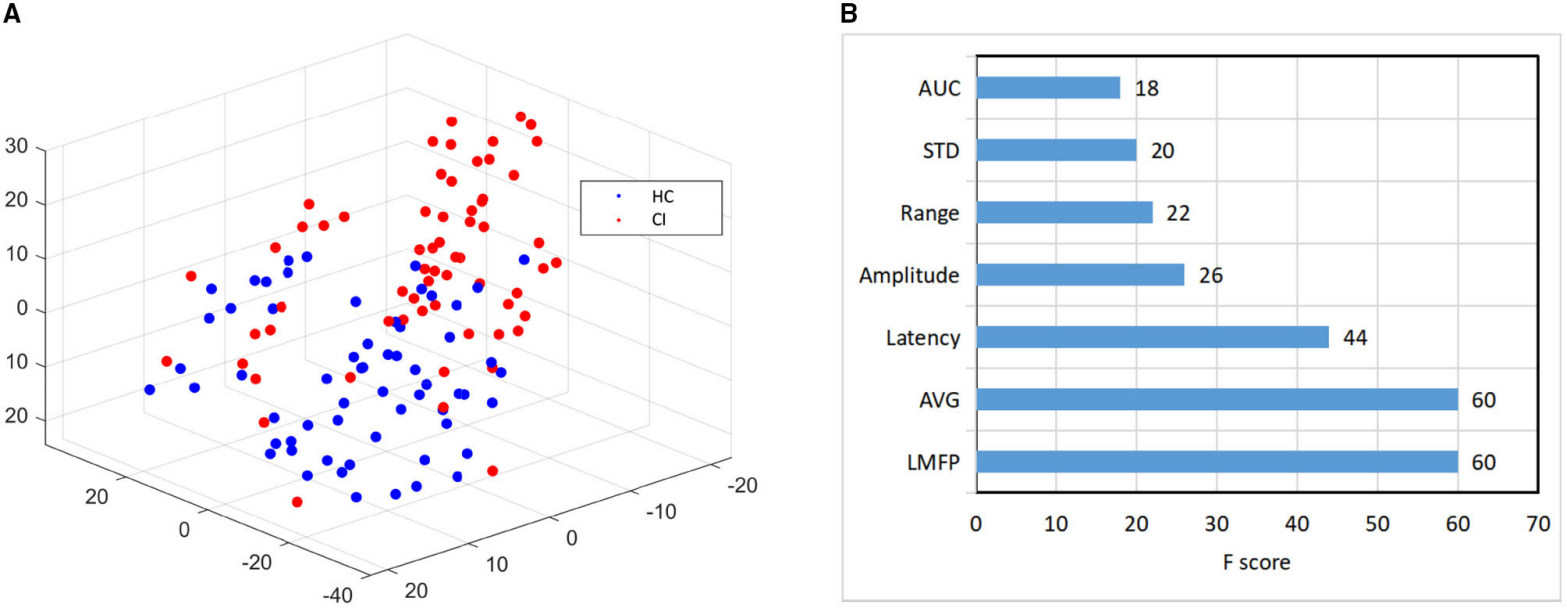
Feature visualization and importance comparison. **(A)** The visualization features map based on t-Distributed Stochastic Neighbor Embedding (t-SNE) dimension-reduction. **(B)** The feature importance based on XGBoost.

Then, we used the t-SNE to reduce the dimension of the best performing N100 features to 3, and then draw them in three-dimensional space. The result was shown in Figure 5A, red represented CI, and blue represented HC. It revealed that the points in CI were more dispersed than normal.

Finally, for the 7 features of N100 in the right frontal region, we used XGBoost to evaluate the importance of features (Chen and Guestrin, 2016). The feature importance ranking was listed in Figure 5B. The LMFP, AVG, Latency, and Amplitude were the most important, which exactly were consistent with the most widely used features to measure TEPs in previous studies (Tremblay et al., 2019). Our results verified the conclusions of the predecessors and also instructed doctors to pay more attention to these features in future clinical practice.

## 4. Discussion

The study showed that machine learning can effectively identify CI. In the time domain, the features of N100 had the best performance. In the aspect of spatiality, the features of the right frontal region and left parietal region had the best performance. Then, we discussed our results from these two dimensions, and particularly emphasized the influence of sensory contamination within the TEP. Finally, we discussed the limitations of our study.

Previous studies have used TMS to stimulate the motor cortex and have demonstrated that patients with AD have increased cortical excitability, which is manifested by a decrease in RMT and an increase in MEP (Alagona et al., 2001; Di Lazzaro et al., 2004). There is also evidence of high excitability in patients with AD after TMS stimulating the left DLPFC, which is manifested as an increase in CEA, and this increase is negatively correlated with overall cognitive and executive functions (Joseph et al., 2021). In our study, the CI group had higher GMFP, suggesting higher cortical excitability which is consistent with previous studies. This hyper-excitability may reflect the plastic reorganization of the sensorimotor system and may be used as a compensatory mechanism to offset the loss of cortical volume and protect the motor function of the patient (Niskanen et al., 2011; Bagattini et al., 2019).

It was found in a previous study that the reliability of the potentials induced by TMS in the left DLPFC are highly consistent and the measurement error is small. The most reliable peaks are generally located at 100 and 200 ms (Kerwin et al., 2018). High reproducibility is necessary as a potential neurobiomarker. Although there was some prior knowledge that CI may affect P30 (Bagattini et al., 2019), considering the first peak may be affected by preprocessing (–5–15 ms removed and cubic interpolation), we still focused on the N100 and P200 two components to preserve the temporal features.

In the time domain, the features of N100 have the best performance according to the four indicators in the classifier. In the CI group, the change of N100 and P200 may be related to the alterations of GABAergic activity. Gamma-aminobutyric acid (GABA) is an inhibitory neurotransmitter. Its natural function is binding to GABA-A receptors and GABA-B receptors on the neurons to modulate and block impulses between nerve cells (Gou et al., 2013). There is some evidence that the amplitude of the TEPs component is related to GABAergic activity. GABA-A receptors agonists (alprazolam and diazepam) and GABA-B receptors agonists (baclofen) can modulate the amplitude of N100 or P200 (Premoli et al., 2014; Murphy et al., 2016). There are also some studies stated that the alterations of GABAergic circuits may contribute to CI by disrupting the overall network function (Li et al., 2016). This may be the neuropathological basis for the difference of N100 and P200 between the two groups, which was the precondition for classification.

In the aspect of spatiality, the features of the right frontal region and left parietal region had the best performance in the SVM classifier. From the statistical results, the right prefrontal region and the left parietal region both had significant differences in these two selected time windows, which may explain why the features of these two ROIs perform better in the classifiers. Interestingly, the significant difference between CI and HC is reflected in the anterior and posterior regions, and the trend is opposite over time. This seems to indicate the abnormal changes in the connectivity of the brains of patients with CI. Previous studies have shown that the damaged excitatory-inhibitory balance between anterior and posterior regions might represent a maladaptive pathogenic mechanism (Bagattini et al., 2019).

In addition, we want to emphasize the issue of sensory contamination within the TEP. There is no doubt that the auditory complex can overlap with N100 and P200 (Conde et al., 2019), but in fact we have used strict online and offline methods to avoid the impact of auditory and somatosensory evoked potential as much as possible. In the data collection process, we played white noise by earphones for the subjects. In addition, a foam layer was placed under the coil to inhibit bone conduction and weaken the scalp sensation caused by the vibration of the coil. During data preprocessing, we paid attention to the removal of auditory artifacts in the second ICA run which were characterized by a topography centering over Cz to Fz and a time course with bipolar peaks at approximately 100–200 ms (Rogasch et al., 2014). From another perspective, all subjects received the same protocol and all the EEG were produced in the same environment and preprocessed by the same method. Therefore, even if the online and offline methods cannot completely remove the auditory and somatosensory evoked potentials, the remaining potentials have the same effect on both two groups, which does not contribute to the difference between groups, let alone the impact on classification. In summary, there is no reason to think that the difference in the selected time windows is related to sensory contamination.

In this study, we observed that all classification results had high specificity but unsatisfied sensitivity, meaning that CI subjects were not well distinguished from HC. It is due to the heterogeneity of cognitive related disease, time of illness, and disease progression may lead to more scattered features. There was a limitation that all patients with CI enrolled were highly suspected of AD with mild to moderate symptoms based on clinical diagnosis and MoCA, excluded vascular and other types of dementia, but not on biological markers of CSF or PET. The definition of AD is controversial throughout articles (Jellinger, 2020; Milà-Alomà et al., 2021). The focus of this study was the extraction method of TEPs' features and machine learning rather than strict AD diagnostic criteria. To be conservative and rigorous, although all subjects in the CI group were highly suspected of AD, we did not define them as AD but summarized it with CI in this study.

A further limitation is that the sample size in this study was small in the field of machine learning. Although we have used data augmentation and voting strategy to obtain a more robust model, more data will still be needed in subsequent studies to meet the real and complex clinical needs.

## 5. Conclusion

We found that the TEPs induced by TMS over the left DLPFC has significant differences between CI and HC. Machine learning based on the spatiotemporal features of TEPs is effective for the classification of CI and HC.

In the time domain, the features of N100 had the best performance in the SVM classifier, with an accuracy of 88.37%. In the aspect of spatiality, the features of the right frontal region and left parietal region had the best performance in the SVM classifier, with an accuracy of 83.72%. By using XGBoost to evaluate the importance of features, the LMFP, AVG, Latency, and Amplitude contributed the most in classification. It is suggested that clinicians should pay close attention to the important features above, which may be potential biomarkers for diagnosing CI.

In this study, the features selected were all simple and linear, the classification algorithms used were popular and sophisticated. Therefore, our research particularly emphasized the interpretability and clinical usability. These findings prove that machine learning based on spatiotemporal features of TEP has the potential to automatically clinical auxiliary diagnosis of CI.

## Data Availability Statement

The raw data supporting the conclusions of this article will be made available by the authors, without undue reservation.

## Ethics Statement

The studies involving human participants were reviewed and approved by the Institutional Review Board of Shenzhen People's Hospital. The patients/participants provided their written informed consent to participate in this study.

## Author Contributions

JZ: writing—original draft preparation, TMS-EEG data artifacts removing, features extraction, and statistical analysis. HL: writing—original draft preparation, machine learning, and feature analysis. LZ: writing—reviewing, conceptualization, and methodology. HR: data collection. XSu and XL: TMS equipment operation and EEG acquisition. GD, XZ, XJ, JF, and XSh: investigation, writing—reviewing, and editing. TW and XH: writing—reviewing and editing, investigation, methodology, and supervision. YG: writing—reviewing and editing, clinical diagnosis, investigation, and methodology. All authors contributed to the article and approved the submitted version.

## Funding

This study was supported by Shenzhen Science and Technology Innovation Commission (KCXFZ20201221173400001 and KCXFZ20201221173411032), Natural Science Fund of Guangdong Province (2021A1515010983). Shenzhen Key Medical Discipline Construction Fund (No. SZXK005), and Special Funds for the Cultivation of Guangdong College Students' Scientific and Technological Innovation (Climbing Program Special Funds, pdjh2021c0018).

## Conflict of Interest

The authors declare that the research was conducted in the absence of any commercial or financial relationships that could be construed as a potential conflict of interest.

## Publisher's Note

All claims expressed in this article are solely those of the authors and do not necessarily represent those of their affiliated organizations, or those of the publisher, the editors and the reviewers. Any product that may be evaluated in this article, or claim that may be made by its manufacturer, is not guaranteed or endorsed by the publisher.
